# Lubricated friction around nanodefects

**DOI:** 10.1126/sciadv.aaz3673

**Published:** 2020-04-03

**Authors:** Clodomiro Cafolla, William Foster, Kislon Voïtchovsky

**Affiliations:** Department of Physics, Durham University, South Road, Durham DH1 3LE, UK.

## Abstract

The lubrication properties of nanoconfined liquids underpin countless natural and industrial processes. However, our current understanding of lubricated friction is still limited, especially for nonideal interfaces exhibiting nanoscale chemical and topographical defects. Here, we use atomic force microscopy to explore the equilibrium and dynamical behavior of a model lubricant, squalane, confined between a diamond tip and graphite in the vicinity of an atomic step. We combine high-resolution imaging of the interface with highly localized shear measurements at different velocities and temperatures to derive a quantitative picture of the lubricated friction around surface defects. We show that defects tend to promote local molecular order and increase friction forces by reducing the number of stable molecular configurations in their immediate vicinity. The effect is general, can propagate over hundreds of nanometers, and can be quantitatively described by a semiempirical model that bridges the molecular details and mesoscale observations.

## Teaser

Nanoscale lubrication measurements around nanodefects point to local molecular ordering as the main cause for friction.

## INTRODUCTION

Friction plays a crucial role in many phenomena, ranging from the molecular diffusion of solutes within cells (*1*) and the function of our joints (*2*) to industrial machinery, car engines (*3*), and the tectonic dynamics (*4*). The ubiquitous importance of friction is perhaps best illustrated by the fact that it accounts for more than 25% of the world’s current energy consumption (*3*). In most systems, friction forces can be markedly reduced by the presence of a lubricant between the moving solid parts. The lubricant is typically arranged in a thin layer confined between the solids and modulates the magnitude and the dynamics of tribological contacts (*3*). However, a comprehensive description of the molecular-level mechanisms underpinning lubricated friction is still lacking (*5*). This is largely due to the fact that our understanding of friction and lubrication is based on semiempirical macroscopic models (*6*–*7*), with no clear or direct links between molecular-level effects and the macroscopic forces they produce (*7*). For lubricating films confined in nanoscale gaps, the behavior of the lubricant tends to be dominated by that of the boundary layer directly in contact with the confining solid (*8*–*10*). This boundary layer behaves very differently than the bulk fluid because of reduced configurational entropy. Furthermore, its interactions with the solid and nanoscale surface singularities can markedly alter the local fluid dynamics. Examples include local molecular segregation (*11*), superlubricity (*9*, *12*, *13*), and cooperative molecular effects (*14*), but the underpinning mechanisms are poorly understood. Molecular-level measurements are challenging (*9*, *11*–*13*, *15*–*18*) and often strongly depend on the precise nanoscale location probed (*9*, *11*, *19*). Since most lubricated systems are imperfect with nanoscale roughness and chemical inhomogeneities at the surface of the solids (*20*), there is a critical need for in situ experimental investigations able to map the molecular details of the boundary layer around surface singularities and directly quantify the local lubrication behavior at the nanoscale.

Here, we combine in situ experiments conducted locally with subnanometer precision and molecular dynamics (MD) computer simulations to investigate the molecular organization and dynamics of nanoconfined squalane near atomic steps of a graphite substrate. Squalane is a branched alkane with six methyl sidegroups (*16*) and is often used as a model lubricant in the formulation of engine oils (*21*) and in pharmaceutical products (*22*). Because of the conformational constraints imposed by the side branches (*23*), squalane does not naturally form ordered layers but exhibits a pronounced interdigitation between adjacent molecules (*24*), enhancing its lubrication properties. Understandably, the nanoscale behavior of squalane as a model lubricant has been extensively investigated with numerous techniques (*11*, *16*–*18*, *23*–*34*), but its behavior under confinement remains a matter of debate. Equilibrium surface force apparatus (SFA) experiments using two atomically smooth mica surfaces as confining solids (*16*–*18*) revealed no oscillatory force profile, suggesting a lack of ordered molecular arrangement. Shearing SFA measurements, however, yielded contradictory results (*17*–*18*), and it is still not clear whether the lubricant molecules can realign along the shear direction or rather along the crystallographic directions of the solid (*16*). In contrast, atomic force microscopy (AFM) measurements routinely identified an oscillatory solvation force profile when confining the squalane between a silicon tip and a smooth graphite surface (*18*, *35*) with at least four layers visible (*35*). The average layer thickness of ~5 Å is larger than would be expected for well-aligned squalane molecule, suggesting confined squalane to behave as an amorphous material with only short-range order. This view is also supported by He atom scattering, neutron scattering experiments, and x-ray reflectivity (*30*, *31*). It should be pointed out that the confinement pressure in AFM measurements is typically six orders of magnitude larger than in SFA because of the highly localized measurement (*35*). In addition, the use of hydrophobic graphite may favor the formation of ordered squalane structures at its interface (*18*). Lamellar structures have indeed been observed by scanning tunneling microscopy (*27*), and ordered structured of nanoconfined asymmetric molecules are also predicted by various theoretical studies based on molecular simulations (*25*, *36*–*38*).

Together, these studies suggest a strong dependence of the squalane behavior on the local nanoscale details of the confinement, with chemistry, geometry, and pressure all playing a role in the molecular arrangement of the lubricant. SFA experiments may average out localized effects (*9*, *18*, *35*), requiring measurements on the same scale as typical surface singularities of solids. To tackle this issue, we conduct high-resolution AFM measurements combining in situ molecular-level imaging and highly localized shearing measurements of squalane near and at atomic steps of highly orientated pyrolytic graphite (HOPG). Our AFM and complementary MD results help disentangle local surface effects from the altered thermodynamics of nanoconfined lubricants at interfaces and provide quantitative molecular-level insights into the impact of surface singularities. We also explore the impact of temperature and shearing velocity on the system to derive a simple molecular model that quantitatively explains the observations.

## RESULTS AND DISCUSSION

### Molecular ordering near surface defects at equilibrium

The simplest and most common surface singularities at the surface of graphite are step edges. To examine the equilibrium organization of squalane molecules near an HOPG step, high-resolution amplitude modulation AFM imaging was conducted with both the imaging tip and the HOPG immersed into squalane. In amplitude modulation, the AFM tip oscillates vertically, and its amplitude is held fixed by constantly readjusting the average tip-sample distance as the tip scans across the sample. These readjustments constitute the topographic information in AFM images. For high-resolution imaging at solid-liquid interfaces, the oscillation amplitude is typically comparable to the thickness of the interface (<1 to 2 nm) (*9*, *39*). The phase of the vibration varies freely and carries information about the local viscoelastic properties of the interfacial liquid (*9*, *39*).

**Fig. 1:**
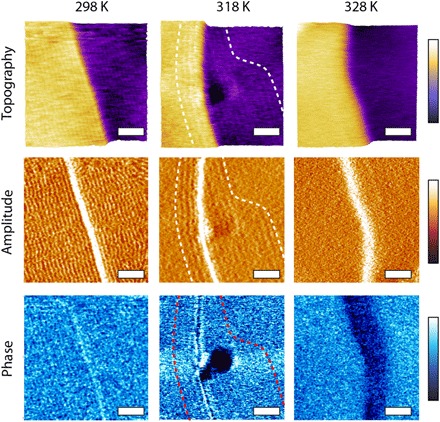
High-resolution amplitude modulation AFM imaging of squalane molecules at the surface of HOPG near an atomic step at 298, 318, and 328 K. Topography, amplitude, and phase images are acquired simultaneously. At 298 K, periodic longitudinal structures running parallel to the step extend over the whole surface and are visible in all three channels. The darker region in phase suggests less mobile squalane molecules (*11*). As the temperature increases, the squalane-based structures progressively retreat to within tens of nanometers of the step (highlighted by the dashed lines at 318 K) to lastly disappear completely at 328 K. Scale bars, 30 nm. The color bars for the topography, amplitude, and phase channels represent variations of 1.0 nm, 0.4 nm, and 2.0°, respectively.

High-resolution imaging of the squalane-HOPG interfaces shows that squalane molecules tend to adopt a row-like equilibrium arrangement near step edges at room temperature (Fig. 1; see also figs. S1 to S3). The arrangement is present both at the top and the bottom of the step but tends to progressively vanish when moving away from the step or when the temperature is increased, here, above 323 K (Fig. 1). This result is consistent with the squalane molecules progressively becoming more mobile away from step edges, rendering the ordered row-like molecular organization either invisible over the time scale of the AFM measurement (~100 s per image) or destroying it together.

### Spatial extent of the influence of defects on lubricated friction

**Fig. 2:**
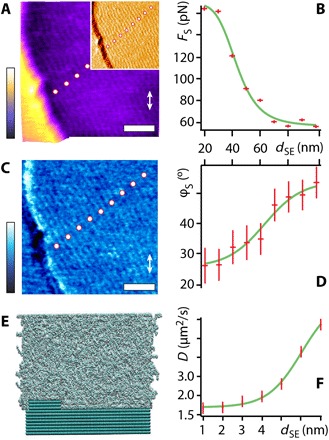
Shear behavior of squalane molecules near an HOPG step edge at 298 K. The row-like arrangement parallel to the edge is visible over the whole AFM images (**A** and **B**). The inset in (A) shows variations in the scanning amplitude where the contrast over the rows is clearer. Shear force spectroscopy measurements taken at set distances *d*_SE_ from the step reveal a decrease in lubricated friction force (shear force *F*_S_) (**C**) and increase in the shear phase φ_S_ (**D**) when moving away from the step. *F*_S_ and φ_S_ are taken at an applied lateral force *F*_L_ ~ 30 nN. The shear direction is illustrated by the white double-headed arrows (A and C). Consistently, MD simulations (unit cell of 34.1 nm ×7.2 nm ×11.8 nm with ~3000 squalane molecules) (**E**) show an increase in squalane diffusion constant, *D*, when moving away from the step edge at the interface (averaged within 1.2-nm layer above the HOPG surface) (**F**). Quantitative comparison between AFM and MD measurements is difficult because of limited size of the simulation box (see fig. S5). Scale bars, 25 nm. The color bars in (A) and (C) represent height variations of 1.2 nm (0.6 nm for inset) and phase variations of 3.0°, respectively.

To get a quantitative dynamical picture of the lubricant, it is necessary to go beyond high-resolution imaging of the interface at equilibrium. To achieve this, we conduct highly localized shear measurements of the nanoconfined squalane molecules along the interface. This is achieved by using the AFM as a nanoscale linear shear rheometer with the tip oscillating laterally at a set distance from the surface. Using small shear amplitudes (~0.5 nm) and the nanopositioning system of the AFM to place the tip, it is possible to conduct highly localized shear measurements at any desired location visible in the high-resolution image (*9*, *11*) (see Materials and Methods for details). Results of the shear measurements are presented in Fig. 2 (C and D) as a function of the distance from the step edge, *d_se_*. The shear force *F*_S_ is an absolute measurement of the average lubricated friction experienced by the tip, and the shear phase φ_S_ quantifies the viscoelastic properties of the confined lubricant (not to be confused with the imaging phase). A value of φ_S_ = 0° indicates a purely elastic behavior of the sheared squalane layer, whereas 90° corresponds to a purely viscous behavior. With a confinement area of ∼20 nm^2^ (see fig. S4 for a detailed analysis on the AFM tips), *F*_S_ can be expressed also as a function of the confining pressure (*9*, *35*): An increase of 1 nN in the applied load increases the confining pressure by ~50 MPa. This makes the present setup suitable to probe the lubricant film in the high-pressure regime typical of mechanical applications (*3*).

Punctual shear measurements taken at different distances from the step edge show a progressive reduction in *F*_S_ and an increase in φ_S_ when moving away from the edge, both consistent with the hypothesis of squalane molecules becoming progressively more mobile and hence less able to resist the applied shear (Fig. 2, C and D). The transition occurs over a relatively large distance (~100 nm), ruling out any tip or convolution effects at the step. This demonstrates that the increase in lubricated friction in proximity of a step edge is not a direct geometrical effect of the tip meeting a rougher area but rather the indirect result of a localized molecular ordering induced by the surface features of HOPG. Shear measurements conducted directly on the step edge show that lubricated friction force is no larger than on the well-ordered adjacent molecular domains, demonstrating that direct tribological contacts with the step and the tip do not dominate the friction (see fig. S2). Row-like domains can be found both at the bottom and at the top of the step edge, with a symmetrical behavior of the lubricated friction as a function of distance from the singularity (see fig. S3). This confirms that the molecular order is a general consequence of the existence of surface singularities and not specific to molecules being stabilized by the edge at the bottom of the step.

The measurements were conducted with diamond-like carbon (DLC)–coated AFM probes to ensure a system as symmetrical as possible with the model lubricant confined between two hydrophobic surfaces (*40*). This ensures that molecular ordering of the lubricant is not due to a significant difference in the affinity between the squalane molecules and the confining surfaces. The use of DLC tips also offers other advantages over standard hydrophilic silicon tip (*35*) such as a high wear resistance and better reproducibility of the shear measurements (*41*). Measurements with DLC-coated tips also better represent tribological contacts given the wide variety of DLC-coated components in mechanical systems (*40*–*41*).

**Fig. 3:**
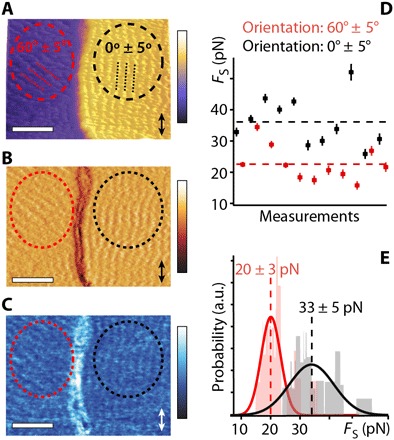
Impact of the molecular ordering of squalane on the lubricated friction force. High-resolution topographic (**A**), phase (**B**), and amplitude (**C**) AFM images of the molecular arrangement of squalane at the interface with HOPG near a step. Domains with different row orientations are visible (dashed red and black circles). Shear force measurements show a clear sensitivity to the rows orientation with statistically higher *F*_S_ values when shearing parallel to the rows (**D** and **E**). Multiple measurements taken over the two regions (A to C) statistically confirm the dependence on the rows orientation (E). Scale bars, 25 nm. The imposed shear direction is indicated with a double-headed arrow (A to C). The dashed lines in (D) and (E) represent mean force values. The color bars represent total variations of 5.0-nm height (A), 0.2 nm (B), and 10.0° (C). The shear forces in (D) and (E) are taken at an applied load of *F*_L_ of 12 nN, and the measurements were performed at 298 K. a.u., arbitrary units.

The behavior observed experimentally at the HOPG step edge could be qualitatively reproduced using MD simulations, albeit on smaller distances from the step due to computational costs (34.1 nm ×7.2 nm ×11.8 nm simulation box with ~3000 squalane molecules and 8 layers of graphene; Fig. 2E). Despite being limited in size and duration, simulations offer a precious point of comparison for the AFM measurements because they are conducted without the AFM tip being present. Since the tip both imposes the confinement and quantifies its effect, examining the behavior naturally adopted by unconfined squalane molecules near the step edge validates the interpretation of the molecular mechanisms at play in the experimental measurements. In addition, MD simulations allow the investigation of the step edge itself, a region difficult to unambiguously probe with AFM because of tip convolution effects, which could potentially affect the results. To quantify the translational mobility of the squalane molecules, we calculated their average diffusion constant, *D*, averaged more than a 1.2-nm-thick band above the HOPG surface (Fig. 2F). A significant increase in *D* can be observed from ~5 nm from the step edge and growing beyond the *d*_SE_ distances simulated here. The full spatial extent of the effect cannot be probed by MD simulation because of the finite size of the simulation box (see fig. S5), but the MD results clearly confirm the fact that squalane molecules move more freely away from the step. The threefold increase in the squalane diffusion coefficient matches remarkably well the magnitude of the reduction in *F*_S_ measured with AFM. The simulations also show an increase in the lateral layering of the squalane molecules close to the step edge, confirming the AFM observation that surface singularities promote molecular order (see also fig. S6). Furthermore, the simulations show that the average orientation of squalane molecules tends to be aligned to the step edge when in close proximity but progressively lose this alignment at larger distances with no clear ordering for *d*_SE_ > 6 nm (fig. S6A). This step-induced molecular orientation is consistent with the row-like features observed in AFM image (Figs. 1 to 3 and figs. S1 to S3).

Overall, both the AFM and MD results show that a reduction of the lubricant’s configurational entropy near surface singularities could provide an alternative molecular mechanism for the well-known increase in roughness-related friction routinely observed macroscopically between two solid surfaces (*42*–*44*). In this framework, the roughness-induced increase in friction around surface defects could be an indirect effect in lubricated systems, a result of molecular ordering in the vicinity of surface singularities, and not dominated by direct solid-solid contacts at singularities. In such systems, the roughness of a solid would determine the density of singularities but not directly the friction force itself. Here, the more ordered squalane molecules have a reduced mobility, inducing larger lubricated friction force. The generality of this mechanism remains to be fully explored beyond the present system and over different spatial scales, but the findings are not restricted only to step edges on HOPG. Lubrication experiments conducted with squalane nanoconfined between an amorphous silicon nitride AFM tip and a molybdenum disulfide (MoS_2_) substrate reveal a comparable behavior (fig. S7). Similarly, mesoscale experiments taken over larger areas of the graphite’s surface show a clear correlation between the apparent surface roughness and the resulting lubrication force despite the tip shearing over a variety of singularities (fig. S7). However, the nanoscale AFM results cannot fully address the link between molecular-level effects and macroscopic friction, and complementary results also involving other techniques (*19*) are needed.

### Impact of the precise molecular ordering around defects

The dominating role of the defect’s “vicinity regions” in modulating the lubricated friction makes them particularly important to understand and model the process at the molecular level. Before examining the dynamical behavior of the lubricant molecules in these regions, we explored the impact of the local equilibrium molecular arrangement by performing shear measurements at locations exhibiting different row orientations around a same step (Fig. 3, A to C). These regions could occasionally be found at lower temperature due to epitaxial effects on the HOPG and are typically orientated at 30° from each other (Fig. 3, A to C; see also figs. S1 and S2). This also confirms the existence of row-like molecular arrangements both at the top and the bottom of the step near the edge (see also Fig. 1 and figs. S1 to S3). When shearing parallel to the row-like features, the tip must disrupt a coherent molecular assembly along its whole path, whereas shearing at an angle allows the tip to “section” more easily the row-like features, hence limiting the experienced *F*_S_ (Fig. 3D). No significant differences in φ_S_ were observed (fig. S8), but a viscoelastic response is always present, confirming that the AFM tip probes the shear response of the confined lubricant and not of the surface itself. These results confirm the importance of the vicinity regions to modulate friction, with the equilibrium molecular arrangement alone able to decrease *F*_S_ by more than 50% (Fig. 3, D and E).

### Impact of shearing velocity and temperature on the lubrication force

**Fig. 4:**
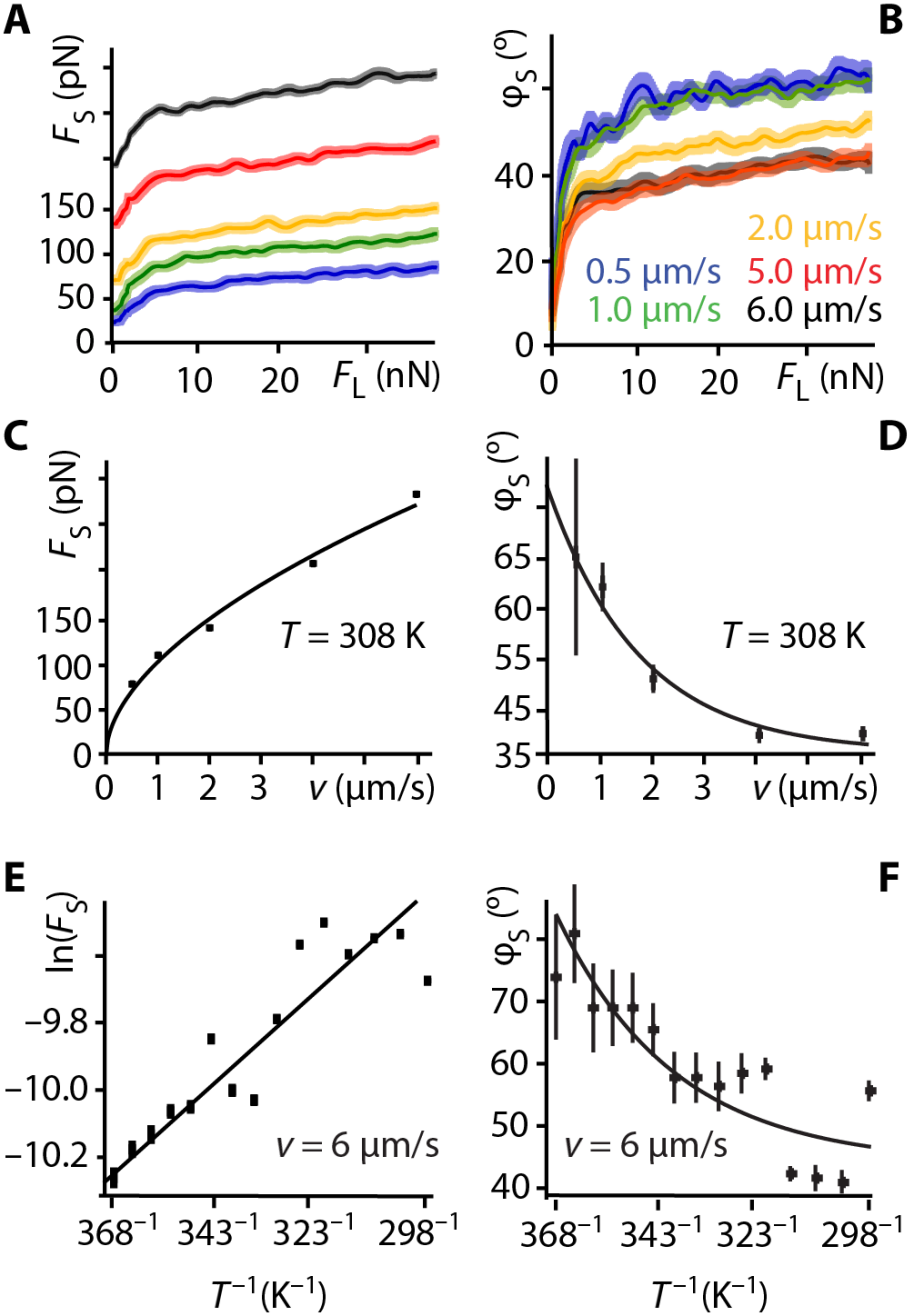
Impact of the tip velocity *v* and the system’s temperature *T* on the lubricated friction response. Representative shear force spectroscopy curves taken at 308 K for different shearing velocities (**A** and **B**) systematically show a two-regime behavior of *F*_S_ with the applied load *F*_L_. As *F*_L_ increases, *F*_S_ exhibits an initial rapid increase akin a yield stress, followed by a more regular plateau-like regime. The associated φ_S_ confirms the existence of a yield stress with a rapid transition from an initial elastic-like behavior to a viscoelastic regime for larger *F*_L_ values (B). In the plateau-like regime, the evolution of *F*_S_ with *v* can be described by a power law at a given temperature (here, 308 K) (**C**). φ_S_ decreases monotonically as a function of *v* (**D**). For a given velocity (here, 6 μm/s), varying temperature shows *F*_S_ to follow an Arrhenius-like behavior with a linear dependence of ln(*F*_S_) versus 1/*T* (**E**). Consistently, φ_S_ shows an increase in viscosity with temperature (**F**). The values of *F*_S_ and φ_S_ are taken at *F*_L_ = 30 nN in (C) to (F).

Lubricated friction is, however, inherently a dynamical process, and insights into the local molecular dynamics are needed to capture the underpinning mechanisms at play. To quantitatively tackle this issue, we exploited the two distinct experimental handles on the lubricant’s molecular dynamics offered by our setup: the average shear velocity *v* of the AFM tip and the temperature *T* of the system. Both variables modulate the relaxation dynamics of the molecules under shear and hence the magnitude of *F*_S_. Representative results showing the evolution of *F*_S_ with the applied load, *F*_L_, are presented in Fig. 4 (A and B) for different *v* values at *T* = 308 K. Similar curves can be obtained when varying temperature at a given shear velocity.

The curves exhibit a first regime characterized by a rapid increase in *F*_S_, followed by a second regime where it reaches a plateau that increases slowly with *F*_L_ (Fig. 4A). The initial increase plays the role of a yield stress, *Y*_0_, likely induced by the local destruction of the lubricant equilibrium molecular arrangement with the tip having to overcome strong cooperative interactions between the squalane molecules. This interpretation is also supported by φ_S_ exhibiting a close to elastic behavior during the yielding process (Fig. 4B). The yield stress depends strongly on the shear velocity and temperature, as to be expected for the activated process of intermolecular bonds rupture (*45*). As *F*_S_ further increases, the second plateau-like regime emerges, with a weak dependence on the applied load and a more viscous behavior (Fig. 4, A and B). When in this second regime and for a given applied load, *F*_S_ increases monotonically with the tip velocity, and the evolution can be empirically captured by a simple power law with an exponent 0 < α < 1 (Fig. 4, C and D). This type of dependence on *v* has been previously reported for similar experiments conducted in other systems (*46*). Here, it implies that *F*_S_→0 when *v*→0, characteristic of the observed smooth sliding in this regime, with no apparent instabilities that could lead to a nonzero value for the low-velocity kinetic friction (*9*, *47*). Generally, the fact that *F*_S_ increases with *v* for all values of *F*_L_ is consistent with the idea that faster velocities allow less time for configurational relaxation of the sheared squalane molecules. This is also supported by φ_S_, which becomes more elastic with increasing velocity because of a frustrated molecular relaxation (Fig. 4D). Generally, the shear behavior of the nanoconfined lubricant is reminiscent of a modified Bingham model where the motion of the lubricant molecules is monotonic and close to Newtonian past an initial yield stress (*48*, *49*).

When varying *T* for a given *v*, *F*_S_ follows an Arrhenius-like behavior within error, with *F*_S_ decreasing exponentially as *T* increases (representative results are shown in Fig. 4E). Similarly to the experiments conducted at different shearing velocities, the temperature dependence is consistent with the idea of a dynamical friction dominated by the configurational relaxation of the lubricant molecules. Higher temperatures favor increased molecular mobility, faster relaxation time, and hence lower friction, together with a more viscous lubricant behavior (Fig. 4F). As can be expected, no high-resolution images could be obtained above 318 K due to the increased molecular mobility preventing the formation of stable row structures (Fig. 1). This view is also supported by computer simulations and other experimental studies that indicate a melting of the solid interfacial squalane layer in contact with graphite between 325 and 338 K (*27*, *50*).

The relaxation dynamics of the lubricant molecules has a marked impact on the lubricant’s effective viscosity η_eff_ when nanoconfined, as to be expected from previous studies (*9*, *15*, *18*). Here, we find an increase of at least three orders of magnitude for η_eff_ at 308 K when compared with the bulk viscosity of squalane, regardless of the applied pressure (fig. S9). However, η_eff_ converges exponentially toward a maximum value for pressures larger than ~0.5 GPa, consistent with the existence of solid-like ordered molecular domains of lubricant molecules. Piezo-viscous effects are also more pronounced for tip-surface gaps smaller than 4 nm (see fig. S9).

### A simple quantitative model

Together, these shearing experiments confirm the dominating role played by the lubricant’s molecular relaxation and provide clues to modeling its behavior. Building on this information, we systematically explored the impact of temperature and shear velocity on the resulting lubricated friction force, probing 14 different temperatures (between 298 and 368 K) and at least five different shear velocities at each temperature. For each measurement, at least 20 individual curves were acquired and subsequently averaged to ensure reliability of the results. The goal is to exploit this comprehensive set of measurements to develop a semiempirical model able to quantitatively describe the dynamical response of the sheared lubricant in all our experiments. To simplify as much as possible the modeling and avoid any overfitting, we fitted the linear region of the shear force curves (*F*_L_ ~10 to 30 nN; Fig. 4A) with a first-order polynomial. This strategy has two advantages. First, it exploits the most reproducible part of the data and extracts a single effective dynamical friction coefficient μ_d_, which can be understood as plastic viscosity in the modified Bingham plastic model. Second, it bypasses the difficulties associated with modeling the yield stress part of the curve (*F*_L_ ≲ 10 nN) where the shear curves tend to present irregular features and shapes. A poor reproducibility at lower loads is inevitable given the stochastic nature of intermolecular bond rupture and possible variations in the local nanoscale details of the squalane/tip system during the initial stages of the shearing. Using the proposed linear fitting, *Y*_0_ (*T*,*v*) is simply the ordinate at origin

$$F_{S}=Y_{0}(T,\nu )+\mu_{d}\;F_{L}$$ (1)

**Fig. 5:**
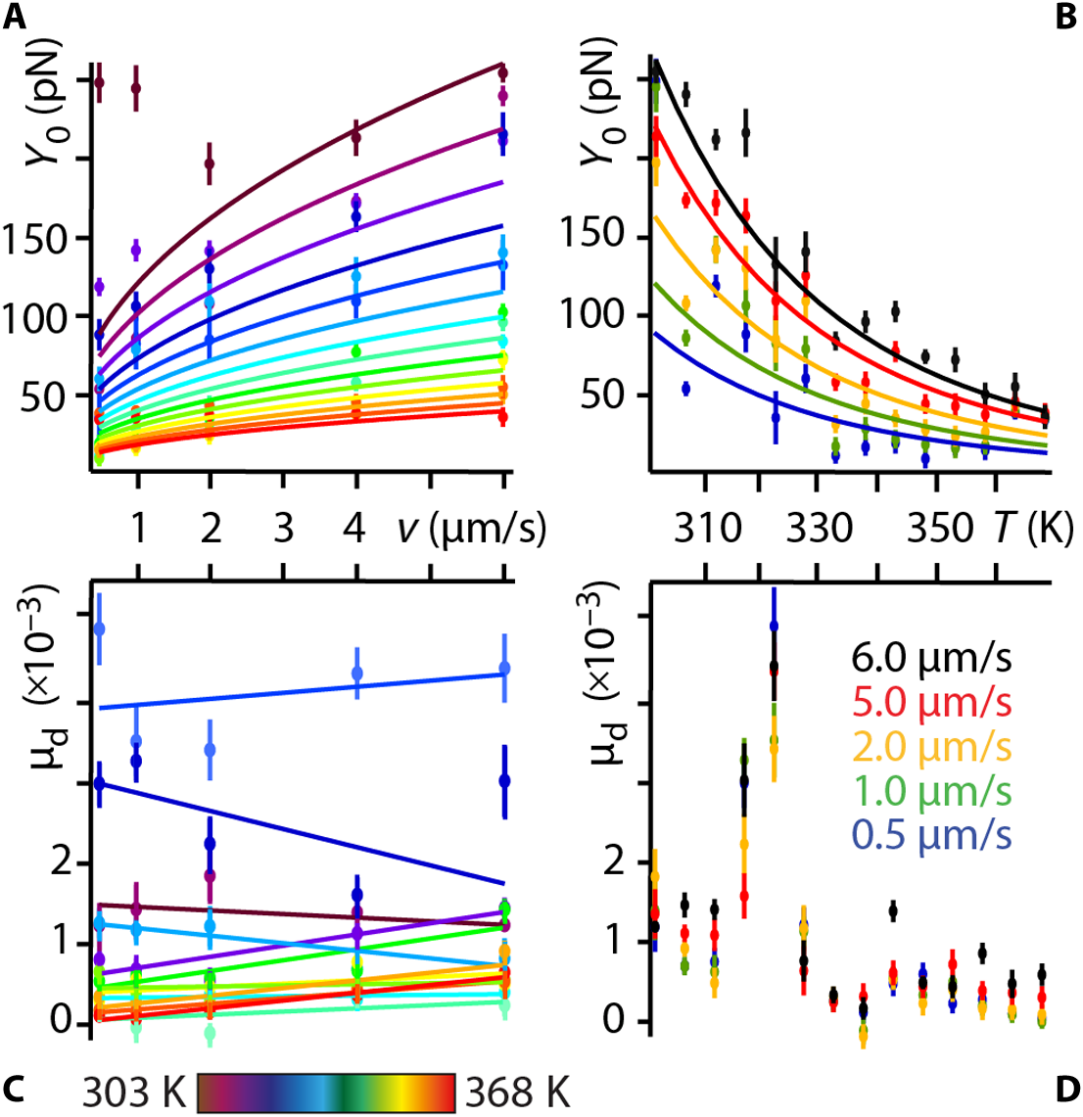
Global analysis of the impact of *T* and *v* on the yield stress, *Y*_0_, and the effective friction coefficient, μ_d_. It is possible to globally fit *Y*_0_ over the whole range velocities (**A**) and temperatures (**B**) probed using Eq. 2. The residuals show no systematic error (fig. S10) with significant outliers only at lower temperatures where interlayer digitation may affect the nanoshearing measurements (*24*). Consequently, the data points at 298 K have been excluded. No clear dependence of μ_d_ on *v* (**C**) and *T* (**D**) is observed within experimental error, except for a local maximum around 323 K, which is ascribed to local melting instabilities in the confined squalane layer (*27*, *50*). Linear fitting of the data at each temperature yields no systematic trend (C). The same fitting coefficients are used for all the fits: *A* = 1.0 ± 0.2 × 10^−12^ (kg/s)^−1/α^, α = 0.43 ± 0.02, and *E*_a_ = −4.46 ± 0.07 × 10^−20^ J (*E*_a_ ~ −11 *k*_B_*T* at room temperature).

It should be pointed out that the fitting does not make any physical assumption beyond the observed linear behavior of *F*_S_ with *F*_L_ and generally provides good fitting (Fig. 5A) with residuals randomly distributed around the zero (fig. S10). The effective yield stress represents an energy-activated process (*9*), and on the basis of the results presented in Fig. 5, the following dependence on *T* and *v* can be assumed

$$Y_{0}(T,v)={\mathrm{Av}}^{\alpha }\text{exp}(-\frac{E_{a}}{k_{B}T})$$ (2)

where *A* is a proportionality constant, *E*_a_ is the activation energy necessary to break intermolecular bonds, and *k*_B_ is the Boltzmann’s constant. Equation 2 effectively combines the dependences of the *F*_S_ on *T* and *v* evidenced in Fig. 5 and does not require any additional assumptions. Using Eq. 2, it is possible to fit globally *Y*_0_(*T*, *v*) over all the temperatures and velocities measured (Fig. 5, A and B). Considering the small number of fitting parameters and the fact that they are global parameters (identical for all the curves), the quality of the fitting is generally good.

Arguably, *E*_a_ is the most fundamental fitting parameter because it describes the energy necessary to break and make new intermolecular bonds. This activation energy effectively determines the stability of any molecular domains under confinement. The global fitting yields *E*_a_ = −4.46 ± 0.07 × 10^−20^ J/molecule, representing ~ −11 *k*_b_*T* at room temperature (298 K). The negative sign is a consequence of Eq. 2 being formulated as a standard Arrhenius behavior. Here, it implies lower lubricated friction forces at higher temperatures. This is indeed the case (Fig. 5) with higher temperatures reducing the number of stable molecular configurations (*51*) and hence the lubricated friction forces. Within this framework, the motion of the squalane molecules can be understood as a limit case of boundary friction where the energy provided by the AFM tip allows the sheared squalane molecules to overcome *E*_a_ and explore neighboring configurations as in the Prandtl-Tomlinson model (*9*, *47*). The activation energy being about 10 times greater than thermal fluctuations at room temperature, thermal contributions only facilitate the onset of motion, inducing a monotonic decrease of the yield stress with increasing temperature (Figs. 4, C and D, and 5, A and B). This interpretation is also supported by the power law dependence of lubricated friction on velocity (Fig. 4C). In the range of velocities explored here, common analytical models predict a logarithmic dependence of the force on the velocity provided that the thermal energy is sufficient to overcome the initial barrier (i.e., *E*_a_ ≅ *k*_B_*T*) (*46*–*47*, *52*). When this last condition is not fulfilled, a power law *v*^β^ behavior is expected with β = 2/3 (*46*). The present results suggest an intermediate scenario where *E*_a_ is larger than the thermal energy, but the latter is already able to facilitate molecular motion. This is consistent with the observed power law dependence of lubricated friction on velocity with an exponent α = 0.43 ± 0.02, smaller than β.

The magnitude of the activation energy can be compared with squalane’s latent heat of vaporization, Δ*H_v_* = 116.2 kJ/mol (~19.3 × 10^−20^ J/molecule) at 378 K (*53*). Δ*H_v_* represents the energy needed to fully remove a molecule from the bulk liquid and separate it from its neighbor. This is a more stringent process than the shearing-induced breaking of intermolecular bonds so Δ*H_v_* is expected to be larger than *E*_a_, but both quantities can be expected within the same order of magnitude. We find Δ*H_v_* ~ 4*E*_a_, consistent with our interpretation of *E*_a_. Considering the fact that sheared squalane molecules at the interface with HOPG have fewer neighbors than in bulk squalane, the magnitude of *E*_a_ does suggest a local “vaporization” of the sheared molecules once past the yield stress. That is, the magnitude of *E*_a_ may be sufficient to almost fully separate squalane molecules from their neighbors as the tip sets the solid-like interfacial layer into motion. This view also implies that once in motion, the molecules may be able to move individually, a bit like hard metal spheres rolling on a smooth flat surface. This shearing regime corresponds to the linear part of the shear curves (second regime; Fig. 4A) and is quantified by μ_d_. The value of μ_d_ does not depend on *T* and *v* within experimental error (Fig. 5, C and D), except for a local maximum around 323 K that may be ascribed to the local melting of the confined squalane (*27*, *50*). This finding supports the view that once in motion, the nanoconfined squalane molecules move either individually or in a very orderly and coordinated fashion so that the influence of the surrounding on any given molecule is minimal. The magnitude of μ_d_ is also relatively low, consistently with typical friction coefficients observed at the limit between boundary lubrication and hydrodynamic friction (*54*). Given the relatively strong squalane-squalane molecular interactions, a low μ_d_ value also supports the existence of a priviledged shearing regime with partilculary low friction. It should, however, be noted that the present work probes a limited range of temperatures and velocities; bulk squalane is liquid between 235 and 449 K at ambient pressure. It is therefore possible for μ_d_ to exhibit a stronger dependence on *T* and *v* outside the range probed here.

## DISCUSSION AND CONCLUSIONS

In this work, we investigate the molecular details of lubricated friction in a well-established model system, squalane nanoconfined over a graphite surface. Combining high-resolution AFM imaging and subnanometric shear force spectroscopy, we quantify the behavior of squalane over a range of temperatures, shearing velocities, and degrees of confinement. The results, supported by MD simulations, show that surface features of the confining solids such as atomic steps locally organize the squalane lubricating molecules in their vicinity due to a loss of configurational entropy around the surface defects. These ordered regions can extend hundreds of nanometers away from the defect depending on the system’s temperature, and the molecular ordering progressively vanishes as the distance from the step edge increases. Shear measurements conducted directly on step edges do not show any increase in the lubricated friction, but rather smaller values than on well-ordered domains, confirming the dominating role of the local molecular arrangement in modulating lubricated friction.

Interfacial regions with higher molecular order can markedly increase the shear force because of the reduced molecular mobility. The present results provide a molecular mechanism whereby roughness is able to affect lubricated friction indirectly and not only through the well-known solid-solid tribological contacts. In the context of this mechanism, surface singularities promote molecular order, which, in turn, reduces the molecular mobility of the squalane lubricant, resulting in increased lubricated friction forces by up to 50% in the present system. Further studies will determine the generality of this mechanism and its applicability to other systems.

We derive a quantitative model able to describe the lubrication mechanism over a range of temperatures and shearing velocities. The model parameters, determined by experimental observations, indicate that the main energy cost to lubrication comes from the rupture of the bonds between lubricant molecules that are resting at equilibrium. Once in motion under the shearing solid, the molecules move almost as if isolated and with limited influence from external parameters such as temperature and shear velocity.

The present study offers fundamentally novel insights into lubricated friction, particularly the role played by the surface asperities typically present on all solids. While some of the results are likely to be system specific, the impact of interfacial domains with reduced molecular mobility and the proposed lubrication mechanism seem more general and may help with the rational design of lubricants.

## MATERIALS AND METHODS

### Sample preparation

The experiments were performed on a freshly cleaved HOPG substrate (SPI Supplies, West Chester, PA) mounted on a stainless steel disk using silver paint (Ted Pella Inc., Redding, CA). High-performance liquid chromatography grade squalane with a purity of ≥99% was purchased from Sigma-Aldrich and used without any further purification (St. Louis, MO, USA).

### AFM measurements

Imaging and shear spectroscopy were conducted using a commercial Cypher ES AFM (Oxford Instruments, Santa Barbara, CA, USA), equipped with temperature control. All the experiments were conducted with a DLC-coated cantilevers (MikroMasch HQ:NSC18/HARD/AL BS, Apex Probes Ltd., UK) with nominal flexural spring constant, *k*_f_, = 2.8 (1.2 to 5.5) N m^−1^ and typical tip radii of 38 ± 4 nm (see fig. S4). Each cantilever was calibrated using its thermal spectrum (*55*) and found to have typical stiffness within the nominal range and *Q* factor of 1.1 ± 0.2 in liquid.

Calibration of the torsional cantilever inverse optical lever sensitivity and spring constant, *k*_t_, returned a value for *k*_t_ = 92 ± 2 N m ^−1^ (*56*). All the experiments were conducted at thermal equilibrium. Thermal equilibrium was assumed when the heating/cooling rates of the temperature control system had been constant for, at least, 15 min. Each series of experiments (including imaging and spectroscopy) was repeated, at least, three times so as to ensure reproducibility of the data.

An accurate cleaning procedure was performed so as to remove any possible sources of contamination (see the Supplementary Materials) (*9*). The absence of contaminants was confirmed by subnanometric imaging of the area investigated (*9*, *57*).

#### Imaging

The AFM was operated in amplitude modulation, fully immersing the cantilever tip in liquid. In this mode, the cantilever was acoustically oscillated at a frequency close to its resonance, and the probe oscillation amplitude, *A*, was kept constant. The phase difference, φ, between the driving and the tip oscillation was allowed to vary freely. The ratio *A*/*A*_0_, between the free amplitude, that is, away from the surface and the working amplitude, was optimized so as to keep it as high as possible, with *A* being between 0.8 and 1.5 nm. This allows resolving atomic details of the interface and any mesoscale structure (*39*, *57*).

#### Shear force spectroscopy

Shear force mode allows using the AFM as a nanoscopic linear rheometer (*9*, *11*). The method is described in full details in (*9*). The sample was subjected to a lateral oscillation at 1.1 kHz, that is, below the resonance frequency of the scanner. The amplitude of the oscillation was 0.5 nm, unless otherwise specified. Three main output signals were recorded as functions of the extension of the cantilever’s base (*11*): (i) the cantilever tip vertical deflection; (ii) the lateral torsional amplitude, *A*_t_, of the cantilever; and (iii) the associated phase, φ_S_ (*11*, *15*). The first signal is used so as to derive the confining force exerted by the tip. From *A*_t_, the magnitude of the shear force, *F*_S_, can be extracted as

$$F_{S}=A_{t}k_{t}$$ (3)

with the model holding true for the small torsional amplitudes used here. The phase characterizes the nature of the coupling, with φ_S_ = 0° and φ_S_ = ±90° corresponding to perfectly elastic and perfectly viscous coupling, respectively (*11*).

For each location explored, a minimum of 20 shear force spectroscopy curves were acquired and consequently averaged. The data were analyzed using homemade programs developed in Igor Pro (WaveMetrics, Lake Oswego, OR, USA) and Python. Before and after changing location for force spectroscopy measurements, high-resolution imaging ensured that no shifts of the sample had taken place affecting the area under investigation.

### MD simulations

The simulations were performed using the MD package GROMACS version 2016 (*58*). The squalane molecules and HOPG system were described by an all-atom OPLS (optimized potentials for liquid simulations) force field (*59*). The squalane molecules without any confinement in the HOPG box were found to have a density of 803 kg/m^3^ at 298.15 K, in agreement with the experimental value of 804 kg/m^3^ (*60*). The system was an isothermal-isobaric (NpT) ensemble maintained at 298 K and 1 bar using a velocity-rescale thermostat (*61*) and Parrinello-Rahman barostat (*62*, *63*) with coupling times of 0.5 and 5 ps, respectively. The pressure coupling was semi-isotropic with the box free to fluctuate only along the *z* direction. During the simulations, the HOPG atoms were not allowed to move. All simulations were performed with a 0.002-ps time step. Before use, the squalane box was equilibrated for 20 ns. After combining with the stepped HOPG, the system was equillibrated for 17 ns before collecting the data over a further 12 ns. The diffusion constant was calculated using the Einstein relation by least squares fitting of the mean square displacement of the squalane molecules (*64*). This was performed using a GROMACS built-in package. Different box sizes were tested, and fig. S5 shows representative results. Because of the large size of the squalane molecules compared with the size of the simulation box, the relatively small size of the box induces an overall reduction in the diffusion constant. This does not affect, however, the observed trend of increased molecular mobility away from the step edge, which appears in all the simulations.

## Supplementary Material

aaz3673_SM.pdf

## SUPPLEMENTARY MATERIALS

Supplementary material for this article is available at http://advances.sciencemag.org/cgi/content/full/6/14/eaaz3673/DC1
