# Supplementary material for: Lubricated friction around nanodefects
Source: Sci Adv. 2020 Apr 3;6(14):eaaz3673. doi: 10.1126/sciadv.aaz3673 (PMC7124950; doi:10.1126/sciadv.aaz3673)
Supplement: aaz3673_SM.pdf [file aaz3673_SM.pdf]

Science Advances
AAAS

advances.sciencemag.org/cgi/content/full/6/14/eaaz3673/DC1

Supplementary Materials for

Lubricated friction around nanodefects

Clodomiro Cafolla, William Foster, Kislon Voïtchovsky*

*Corresponding author. Email: kislon.voitchovsky@durham.ac.uk

Published 3 April 2020, *Sci. Adv.* **6**, eaaz3673 (2020)
DOI: 10.1126/sciadv.aaz3673

This PDF file includes:

Sections S1 to S11
Figs. S1 to S10

1. Domain orientation around surface defects

Figure S1 further confirms the formation of domains with different orientations close to surface defects. This is due to the step edge limiting the number of configurations the squalane molecules can explore. Epitaxial effects on HOPG determine the organization of domains at about at 60° from each other.

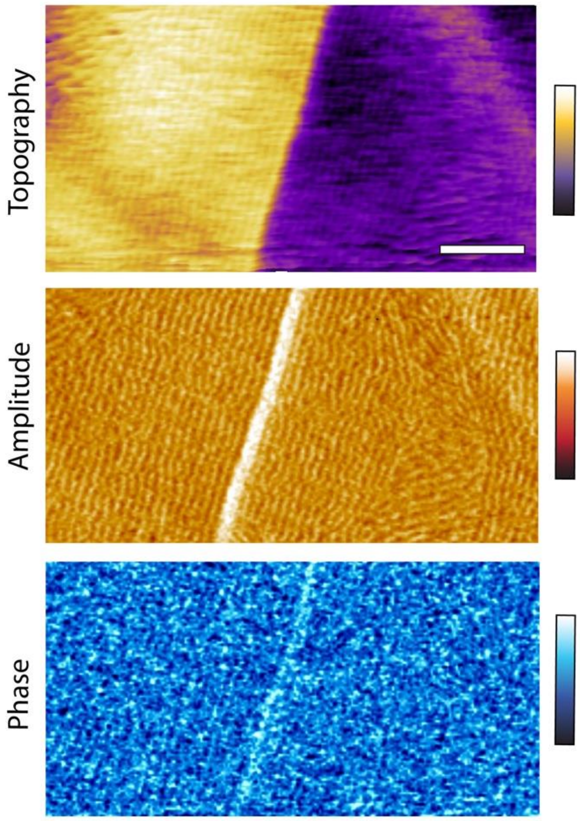

Fig. S1. High-resolution amplitude modulation AFM imaging. *The scale bar is 50 nm in all the images. The color bars for the topography, amplitude and phase channels represent variations of 1.0 nm, 0.4 nm, and 2.0 °, respectively. The experiments were performed at 298 K.*

2. Shear force measurements at and on the top of the step edge

a. Measurements at the step edge

Figure S2 shows well-ordered domains in proximity of the step edge. Lubricant molecules are arranged into rows both at the top and at the bottom of the step edge. Epitaxial effects on HOPG may also contribute to the domains being oriented at about at 60° from each other (Fig. S2 A-C). The different orientations of the domains have a dramatic effect on the lubrication friction force. The lubricant domains oriented parallel to the shearing direction induce the largest shear (Fig. S2 D), larger than directly on the step edge. It should be pointed out that accurately probing the lubricated response on the step edge poses some challenges. Even if thermal stability is ensured (see Methods), there can be some drift of a few nanometers along the direction perpendicular to the shearing, leading to the tip also probing the geometry of the step itself or the ordered region close to the step. However, such problems would tend to increase the measured forces, confirming the dominating role played by molecular ordering.

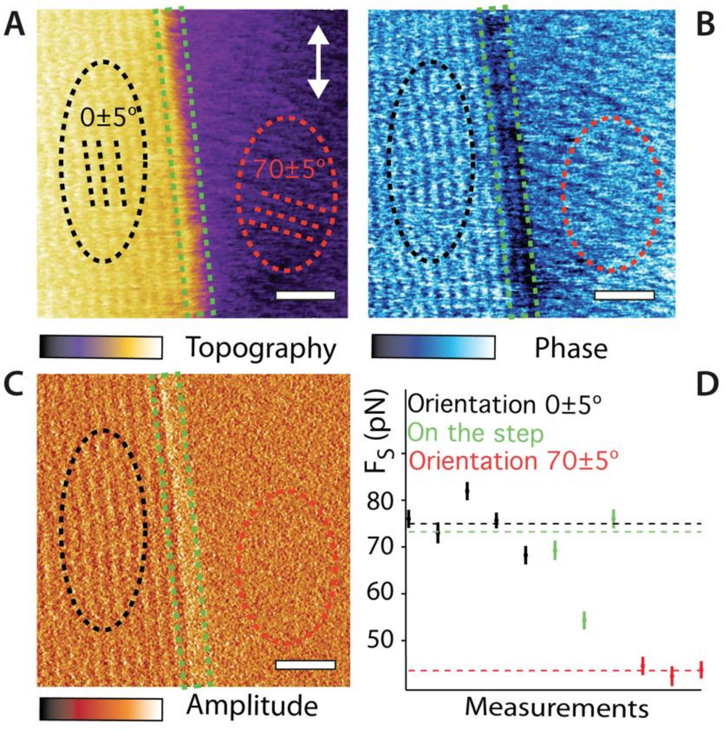

Fig. S2. High-resolution amplitude modulation AFM images. *The topography (**A**), phase (**B**) and amplitude (**C**) images show, around the step edge, different row orientations for the lubricant molecules. The orientations result in significantly different lubricated friction forces as shown in (**D**). The imposed shearing direction is perpendicular to the image as shown by the arrows in (**A**). The scale bar is 20 nm in all the images. The color bars in (**A**), (**B**) and (**C**) represent variations of 1.0 nm, 2.0°, and 0.1 nm, respectively. The experiments were performed at 298 K.*

b. Measurements at the top of the step edge

Molecular ordering is also present at the top of the edge (Fig. S3), confirming that the effect is not restricted to the bottom of the step. Molecular ordering affects the response of the sheared lubricant in a symmetrical way when compared to the scenario at the bottom of the step edge (Fig. 2 in the main manuscript): moving away from the step edge, the lubricated friction exponentially decreases and the shear phase increases (Fig. S3 B and D).

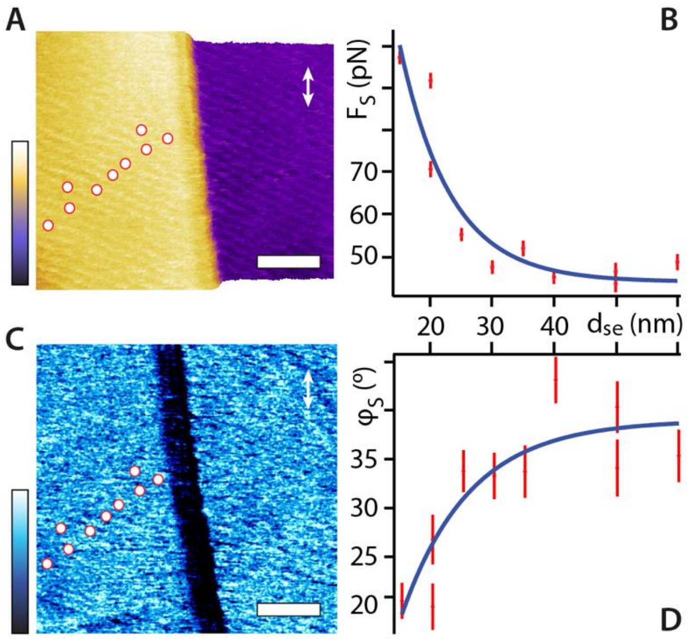

Fig. S3. Molecular ordering of squalane molecules at the top of the step edge. *High-resolution amplitude modulation AFM images (**A, C**) show the organization of the molecules into row-like domains parallel to the step edge. Shear force spectroscopy measurements taken at set distances d_{SE} from the step reveal a decrease in lubricated friction force (shear force F_S) (**B**) and increase in the shear phase φ_S (**D**) when moving away from the step. F_S and φ_S are taken at an applied lateral force F_L ~ 30 nN. The shear direction is illustrated by the white arrow (**A, C**). The color bars in (**A**) and (**C**) represent height variations of 1.2 nm and phase variations of 3.0 ° respectively. The scale bar is 35 nm in all the images. The experiments were performed at 298 K.*

3. Scanning Electron Microscopy analysis of AFM probes

Five cantilevers were randomly selected and imaged using scanning electron microscopy (SEM), before and after conducting the shear-force measurements. The cantilevers were not gold-coated before SEM imaging due to avoid overestimating the apex's size. The SEM analysis was performed with a commercial FEI Helios SEM system (Dawson Creek Drive Hillsboro, Oregon 97124 USA). Images were collected using a 3 kV electron beam in vacuum and the current set to 0.17 nA.

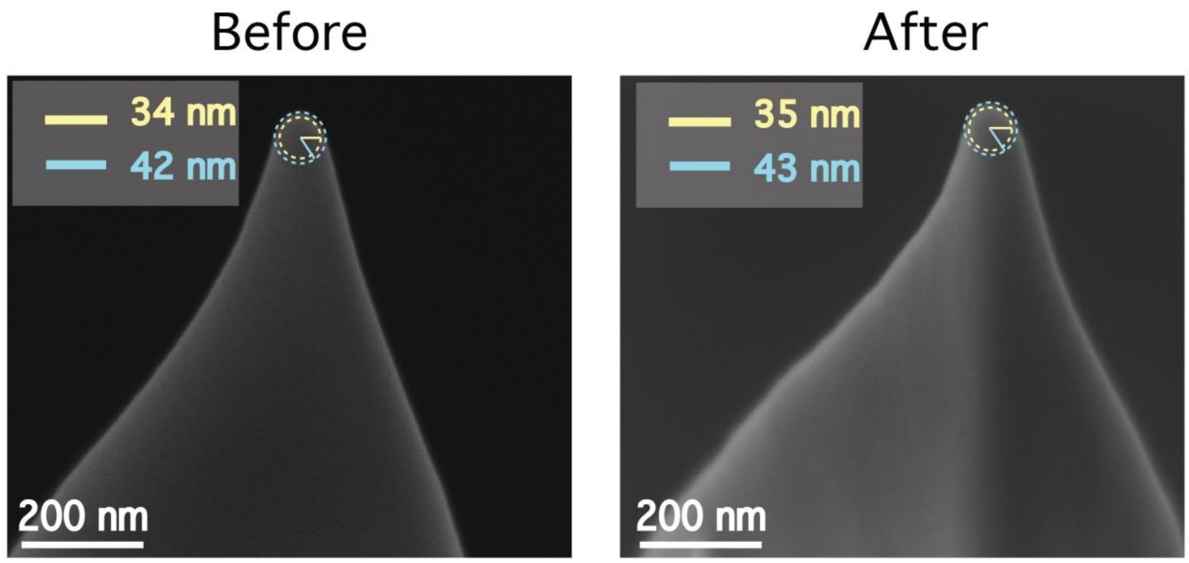

Fig. S4. Representative images of the AFM probes obtained by scanning electron microscopy (SEM) images. *Randomly selected tips were analyzed before and after shear-force measurements, with no significant changes visible. The tip radius is quantified by fitting the tip apex with a circle on the SEM image (dashed circles). To account for apex non-sphericity and uncertainties related to the SEM image quality, two limit cases are considered with a smallest and largest possible circle (yellow and blue respectively). The resulting tip radius value in this image is the average of the extrema: 38 ± 4 nm.*

Representative SEM images are shown in Fig. S4, further confirming the reliability of the shearing measurements with no significant change in their shape or radius after the shearing experiments. Accurate calculation of the tip radius is challenging due to the fact that it is not perfectly spherical and the SEM image imperfect. To take this into account and determine a meaningful uncertainty, fitting of the apex was carried out with two different circles by either placing the weight on the very apex (smaller circle) or on the overall tip (larger circle). The average radius for all the cantilevers analyzed was found to lie between 34 nm and 43 nm.

4. Molecular dynamics simulations: effects due to box size and periodicity

Molecular dynamics (MD) simulations were performed with different box sizes. The results confirm the impact of the vertical confinement on decreasing the mobility of the squalane molecules. As shown in Fig. S5, smaller nano-gaps result in reducing the diffusion constant, *D*. The squalane molecules within Box 1 (red) reach a value for *D* of about 5.5 μm^2/s. Reducing by about 1 nm the size of the box along the z-direction halves *D* (see Box 2 and the corresponding diffusion constant values in blue). The degree of vertical nano-confinement does not affect, however, the trend of increased molecular mobility away from the step edge. This confirms the role of surface defects on limiting the entropy of lubricant molecules. When the distance d_{SE} from the step edge is greater than 4 nm and 9 nm for Box 1 and 2, respectively, *D* shows an apparent decrease. This is due to the periodicity of the MD simulations along the x and y directions. In other words, molecules at the right edge of the two boxes in Fig. S5 are undergoing confinement effects due to another step edge.

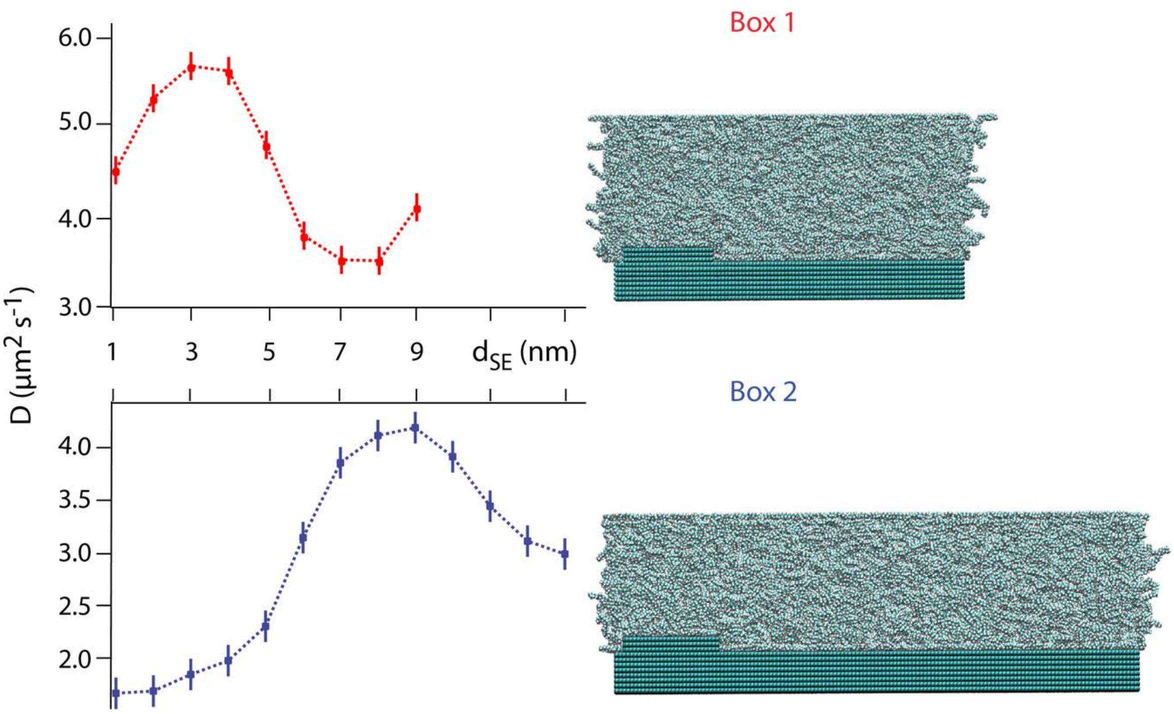

Fig. S5. Effects of box sizes on the diffusion constant, D. *Box 1 and box 2 size are (24.1 × 7.2 × 12.9) nm and (34.1 × 7.2 × 11.8) nm, respectively. The confinement along the z-direction has a clear impact on D, but does not affect the trend of greater molecular mobility as the distance* d_{SE} *from the step edge increases.*

5. Molecular dynamics simulations: ordering, orientation and densification

To investigate the impact of the step edge on the molecular ordering of the squalane, we calculated an orientation order parameter as a function of the distance from the step. The parameter, S_y, is defined as follow (*58*):

$$S_y = \frac{3}{2}\langle\cos^2\theta_y\rangle - \frac{1}{2} \qquad (S1),$$

where θ_y is the angle formed by the y-axis of the step edge (lateral orientation of the step in the plane of the surface) and the molecular axis of squalane. Defined in this way, the order parameter can vary between $S_y = 1$ and $S_y = -1/2$ for an arrangement of the squalane molecule parallel and perpendicular to the step edge, respectively. A value of $S_y = 0$ indicates no preferential order (*58*). Here, S_y is calculated from the squalane molecules whose center of mass lies within 1.2 nm of the surface (~3 molecular layers). As visible in Fig. S6 A, S_y progressively decreases from positive values at the step edge to almost zero past distances d_{SE} > 6nm. This is consistent with the squalane molecules being preferentially oriented parallel to the step edge when in close proximity, with this preferential order progressively being lost away from the step where no molecular orientation is preferred. This confirms that the step edge not only limits the mobility of the squalane molecules but promotes a specific molecular orientation consistent with the row-like domains observed in the AFM images (see also Figs S1-3).

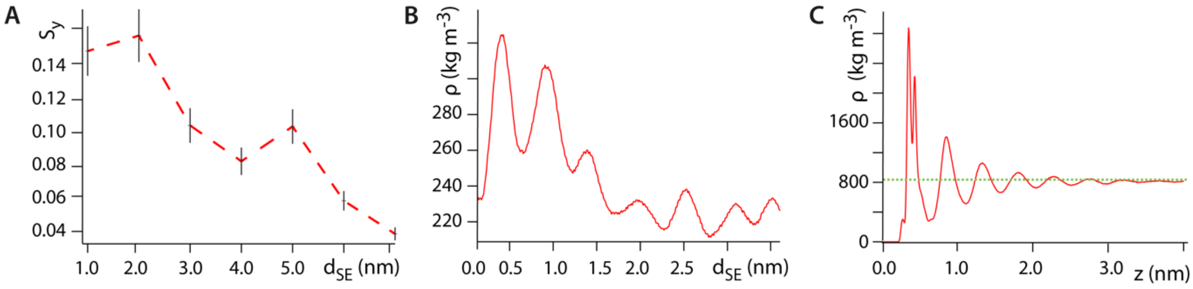

Fig. S6. Average orientation and density profiles of squalane as functions of distance from the step edge (A-B) and from the substrate (C). *In close proximity to the step edge (**A**), squalane molecules are preferentially oriented parallel to the step axis (S_y > 0), favoring the orientation of the row-like domains observed by AFM. As the distance from the step edge increases, the molecules become randomly oriented (S_y ~0). Density profiles taken parallel (**B**) and perpendicularly (**C**) to the surface show the molecular layering. Along the surface (**B**), interfacial effects lead to an oscillatory profile near the step but ρ is noisy due to the limited number of molecules (~3) being probed at each distance from the step. In the vertical profile (**C**) a characteristic oscillatory profile can be observed, progressively vanishing over ~3 nm past which the confined fluid ρ is the*

same as in the bulk (804 kg/m^3, green dotted line). The split peak in the first layer is an artefact due to sampling. Box size is (34.1 × 7.2 × 11.8) nm.

We note that even at the step, the value of the order parameter tends to be small (< 0.2). This is because a value of 1 would indicate the squalane molecules perfectly extended and exactly aligned along the step. In practice, this almost never occurs due to the fact that the squalane molecules are not rigid rods but can bend and accommodate multiple curvatures, especially at room temperature. As such, even molecules visually oriented along the step will fluctuate thermally and see their S_y parameter reduced from 1. However, the clear trend visible in Fig. S6 A and the fact that we consistently have $S_y > 0$ near the step unambiguously indicate a preferred molecular orientation along the step axis.

The lateral and vertical confinement near the step also influence the layering of the squalane molecules with characteristic oscillatory profiles visible, both laterally and vertically (Fig. S6 B-C). This is best seen in the average density profiles taken horizontally (B) as a function of distance from the step edge and vertically (C) from the HOPG surface. The distance between adjacent layers is approximately 0.50 ± 0.05 nm in both (B) and (C). These results are consistent with previous experimental and theoretical observations (26, *32-33*, *38*). The layering is comparable to the width of a squalane molecule, also consistent with the idea that the step edge induces lubricant molecules to arrange parallel to it, flat on the HOPG surface.

6. Impact of other surface defects on lubricated friction

a. Molybdenum disulfide as another test material

We studied the behavior of squalane nano-confined between an amorphous silicon nitride tip (Arrow UHF, Nanoworld, Switzerland) and a molybdenum disulphide (MoS_2) substrate (SPI supplies, West Chester, PA, USA). MoS_2 sheets were attached to a steel plate using epoxy glue (Araldite, Basel, Switzerland) and cured overnight at 65 °C so as to ensure good thermal contact between the MoS_2 and the steel plate for temperature-controlled experiments. The MoS_2 surface was then freshly cleaved with adhesive tape before immersion in squalane for the experiments.

As visible in Fig. S7 A, at room temperature, surface features such step edges and local dips promote molecular ordering of the lubricant just as for HOPG, also with domains forming at both the top and the bottom of a step edge. When the temperature increases, the lubricant molecules become more mobile and molecular domains can no longer be observed (Fig. S7

B), inducing a significantly lubricated friction force than at room temperature (Fig. S7 C). Overall, the experiments indicate that the hypothesis of lubricated friction being indirectly influenced by the surface features is also valid for MoS_2.

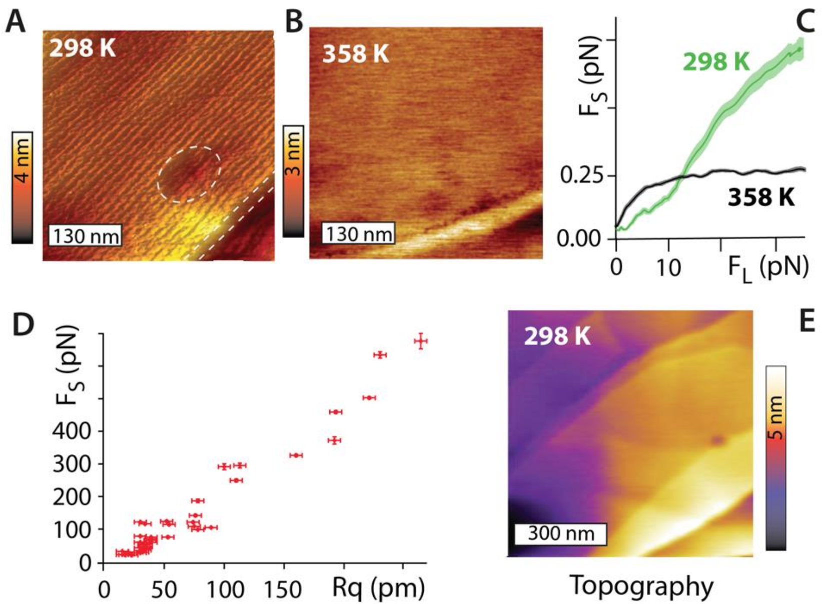

Fig. S7. High resolution imaging and shear behavior of squalane molecules near MoS_2 singularities, and impact of other HOPG singularities on the lubricated friction force. *High-resolution topographic amplitude-modulation AFM images on MoS_2 reveal long-range ordering of the squalane molecules around surface defects at room temperature (298 K) (**A**). The dashed lines highlight some local surface singularities, here a concave depression and a step edge with the ordered squalane structures aligned along the step. When increasing the temperature to 358 K (**B**), no clear molecular details are visible due to an increased mobility of the squalane. Shear spectroscopy measurements (**C**) confirm the impact of the molecular organization on the lubricated friction force. To explore nano-lubrication around HOPG surface singularities other than a step edge, shear spectroscopy was performed on randomly selected locations (**D**) taken across larger areas of the HOPG region as exemplified in (**E**). No molecular order of the lubricant can be seen at this scale but features such as atomic steps or depressions are visible. A clear correlation between the lubrication force and the root mean square roughness is present.*

b. Other surface singularities on HOPG

To explore the impact of surface singularities beyond the well characterized step edge examined in Figs 2 and 3, we surveyed larger HOPG regions, conducting shear force experiments at random locations taken over a wide region (Fig. S7 D-E). The actual shear measurements are conducted in an identical manner to those described in Figs 2 and 3 in terms of amplitude and frequency. However, picking randomly locations across larger areas statistically ensures that a variety of possible surface singularities are included and allows for multiple tip asperities interactions. Given the large regions surveyed, molecular order cannot be seen (Fig. S7 E). The measured lubricated friction force tends to increase almost linearly with the average root mean square roughness of the area probed, supporting the idea of group effects involving defect-induced organized molecular domains (Fig. S7 D).

7. Impact of molecular orientation on the shear phase

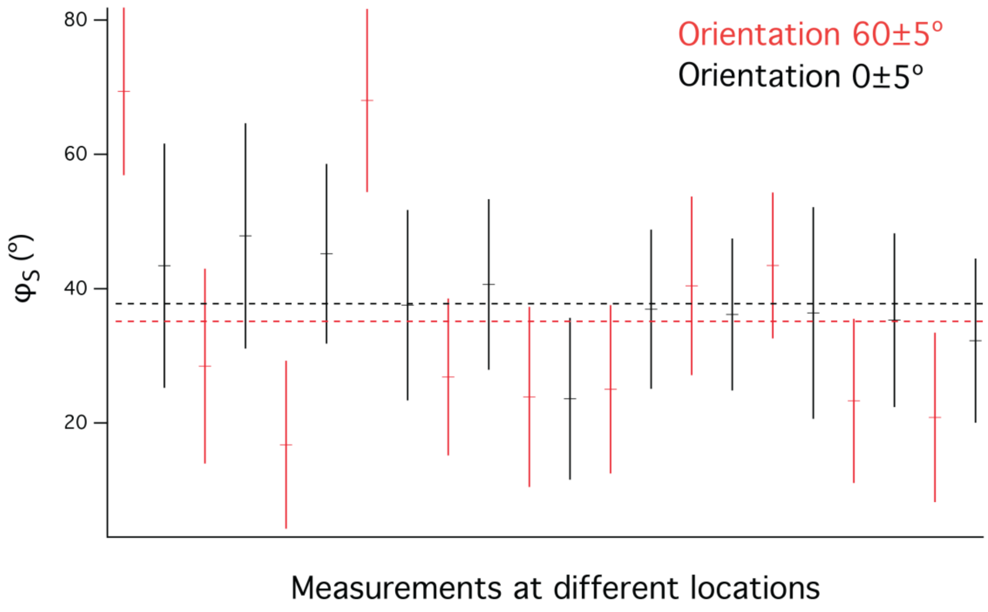

Fig. S8. Shear phase φ_S associated with the lubrication force presented in in Fig. 3. *φ_S values are taken at an applied load of 12 nN. No clear distinction can be seen between the two different row orientations, but the φ_S values indicate that the interfacial lubricant is in a viscoelastic regime.*

8. Calculation of the effective viscosity under confinement

Deriving an accurate value for η_{eff} from shearing experiments requires some knowledge about the shearing boundary conditions, namely the slip length of the lubricant on the surface and the contact area of confinement (*9*):

$$\eta_{eff}(d) = \frac{F_s \sin(\phi_s)\,(d+b)}{A\,v}, \qquad \text{(S2)}$$

where $F_s \sin(\phi_s)$ is the viscous component of the shear force, d the tip-sample distance, b the slip length of the liquid at the interface with mica, A is the tip-sample contact area of confinement, and v the shearing velocity.

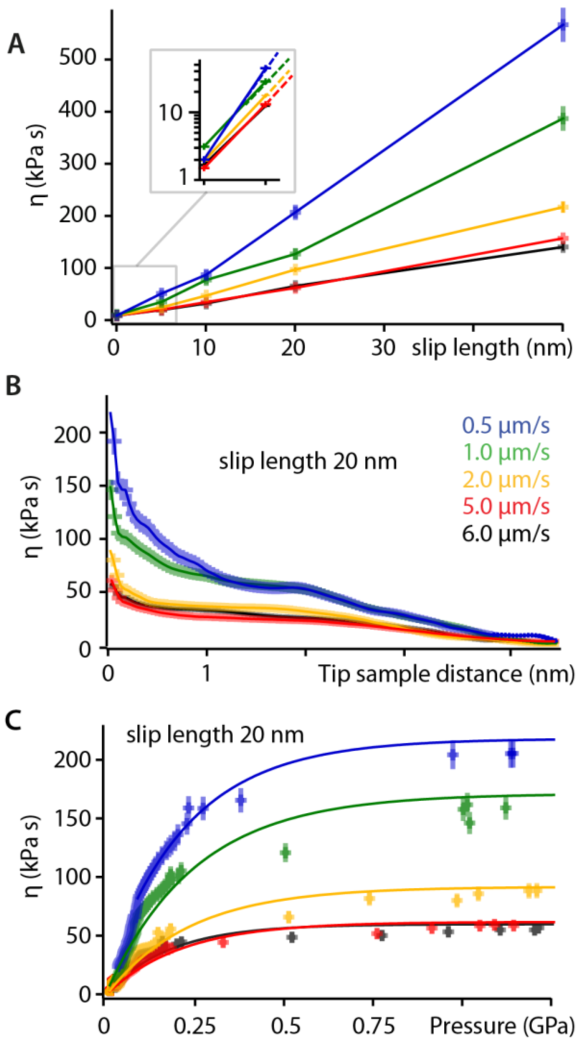

Fig. S9. Pressure dependence of the confined lubricant's viscosity. *Effective viscosity calculated at 308 K and an applied pressure of 1.25 GPa for increasing values of slip length (**A**). The effective viscosity increases by orders of magnitude for tip-sample distance smaller than ~4 nm (**B**). The viscosity shows an exponential dependence on pressure (**C**), reaching a maximum value for confining pressures larger than ~0.5 GPa.*

From electron the microscopy images of the tip (Fig S4), we estimate a typical contact area of ~20 nm^2. The slip length of squalane on HOPG is to the best of our knowledge not known. A no-slip condition would seem reasonable considering the apparent static organization of the lubricant molecules (*9*, *15*), but this approximation may not hold true if molecules are able to escape their surroundings and move along the surface (*48-49*). A slip length of up to ~200 nm has been reported for squalane on sapphire (*34*). To address this discrepancy, we calculated η_{eff} for a range of plausible slip lengths (0 to 50 nm) so as to derive an order of magnitude for η_{eff} (Fig. S9 A). Unsurprisingly, we find higher effective viscosities for smaller shearing velocities.

Representative results calculated assuming a slip length of 20 nm (Fig S9 B) show a significant increase of η_{eff} for gaps smaller than 4 nm, consistent with the molecules arranging in ordered solid-like domains (*9*). This is also supported by the existence of a maximum effective viscosity as the confining pressure increases (Fig. S9 C). Here, the evolution of the viscosity is modelled as an exponential function of the pressure described by the Barus equation (*49*): within this elasto-hydrodynamic model, the continuous film between two sliding surfaces is preserved even at high pressure thanks to an exponential rise in the viscosity. Such a viscosity increase prevents the flow of the fluid outside of the nano-confined gap (*49*). A plateau regime appears for pressures larger than about 0.5 GPa, suggesting that piezo-viscous effects are limited by the molecules displaying more pronounced solid-like characteristics.

9. Global fitting residuals

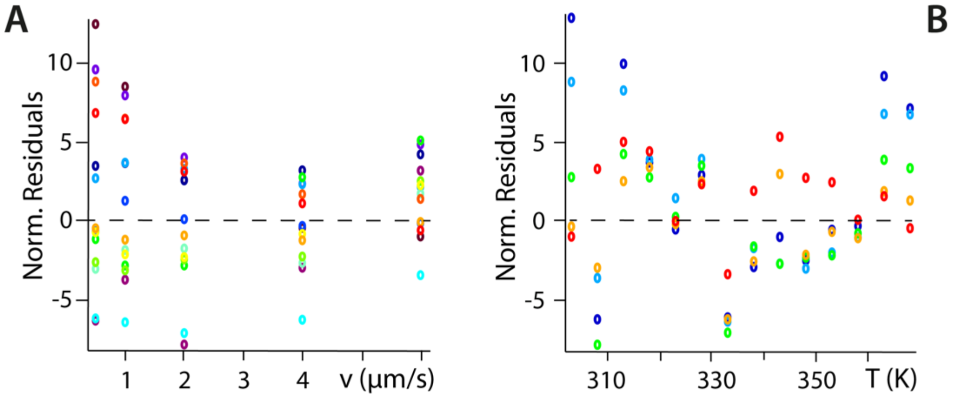

Fig. S10. Normalized residuals for the fits presented in Fig. 5A-B. *The residuals do not show any pattern and appear randomly distributed around the zero, as expected for a suitable model.*

10. General cleaning procedure

Squalane was stored into borosilicate glass bottles (Pyrex, Corning, NY, USA). The glass was cleaned so as to remove any polar and nonpolar contaminant. The cleaning procedure was the following (*9*, *57*): (i) tap water and soap, (ii) rinse with tap water 20 times, (iii) ultra-pure water (18.2 MΩ, Merck Millipore, Billerica, MA, USA), (iv) >98% pure propan-2-ol (Merck Millipore, Billerica, MA, USA), and finally (iv) ultra-pure water. Nitrogen flux was used in order to ensure that no water molecules were present in the dry glass container. The propan-2-ol was used without further purification.

Prior to any imaging and shear spectroscopy, the AFM probes were left soaking in a bath of ultrapure water, followed by propan-2-ol, and finally ultrapure water, for 20 minutes at each step (*9*). The AFM components which were either directly or indirectly in contact with the sample were carefully cleaned using a three-step procedure: ultra-pure water, (iii) >98% pure propan-2-ol (Merck Millipore, Billerica, MA, USA), and (iv) ultra-pure water again. After mounting the cantilever within the AFM stage, the stage was heated up to 378 K for 20 min in order to reduce to a minimum, by evaporation, any possible contaminant traces (*57*).

11. Error analysis

The temperature was considered to be affected by an uncertainty of 0.1 K. Even if the AFM temperature control system is one order of magnitude more precise, we overestimated the error so as to account for errors due to temperature gradients in the highly viscous liquid.

The error on the shear force, shear phase and applied force was the standard error in each case.

Using the z-piezo noise (nominal value < 50 pm), we derived the error on the deflection signal and on the normal force (*9*). The uncertainty on the tip velocity was calculated using standard formulae for error propagation, considering that the uncertainty on the tip oscillation amplitude and frequency are 0.05 nm and 10 Hz, respectively. The error on the natural logarithm of a_0 and a_1 in the Arrhenius plots were calculated using standard formulae for error propagation.

The uncertainty on the diffusion constant is calculated as the standard error.
